# Homologous Recombination Repair Deficiency and Implications for Tumor Immunogenicity

**DOI:** 10.3390/cancers13092249

**Published:** 2021-05-07

**Authors:** Sandra van Wilpe, Sofie H. Tolmeijer, Rutger H. T. Koornstra, I. Jolanda M. de Vries, Winald R. Gerritsen, Marjolijn Ligtenberg, Niven Mehra

**Affiliations:** 1 Department of Medical Oncology, Radboud Institute for Molecular Life Sciences, Radboud University Medical Center, 6525 GA Nijmegen, The Netherlands; sandra.vanwilpe@radboudumc.nl (S.v.W.); Sofie.Tolmeijer@radboudumc.nl (S.H.T.); Winald.Gerritsen@radboudumc.nl (W.R.G.); 2 Department of Tumor Immunology, Radboud Institute for Molecular Life Sciences, Radboud University Medical Center, 6525 GA Nijmegen, The Netherlands; Jolanda.deVries@radboudumc.nl; 3 Department of Medical Oncology, Rijnstate Hospital, 6815 DA Arnhem, The Netherlands; RKoornstra@Rijnstate.nl; 4 Department of Human Genetics, Radboud Institute for Molecular Life Sciences, Radboud University Medical Center, 6525 GA Nijmegen, The Netherlands; Marjolijn.Ligtenberg@radboudumc.nl; 5 Department of Pathology, Radboud Institute for Molecular Life Sciences, Radboud University Medical Center, 6525 GA Nijmegen, The Netherlands

**Keywords:** cancer, homologous recombination repair deficiency, immune checkpoint inhibitors

## Abstract

**Simple Summary:**

Cells possess several pathways that repair DNA damage. One of these pathways is homologous recombination repair (HR), a pathway responsible for the repair of double-strand DNA breaks. In cancer, HR is sometimes dysfunctional, leading to genomic instability. The genomic instability observed in HR-deficient (HRD) tumors has been suggested to alter immunogenicity and render these tumors more susceptible to immunotherapy. In this review, we summarize the available evidence for an association between HRD and tumor immunogenicity. Although there are indications for increased efficacy of checkpoint inhibitors in HRD tumors, data from prospective studies is needed to validate whether HRD can function as a biomarker for patient selection. The extensive overview provided here can be used to guide further research in the field.

**Abstract:**

Homologous recombination repair deficiency (HRD) can be observed in virtually all cancer types. Although HRD sensitizes tumors to DNA-damaging chemotherapy and poly(ADP-ribose) polymerase (PARP) inhibitors, all patients ultimately develop resistance to these therapies. Therefore, it is necessary to identify therapeutic regimens with a more durable efficacy. HRD tumors have been suggested to be more immunogenic and, therefore, more susceptible to treatment with checkpoint inhibitors. In this review, we describe how HRD might mechanistically affect antitumor immunity and summarize the available translational evidence for an association between HRD and antitumor immunity across multiple tumor types. In addition, we give an overview of all available clinical data on the efficacy of checkpoint inhibitors in HRD tumors and describe the evidence for using treatment strategies that combine checkpoint inhibitors with PARP inhibitors.

## 1. Introduction

Cells possess a complex set of non-redundant and partially overlapping pathways to detect and repair DNA damage, including base modifications, strand breaks, and interstrand crosslinks. Major DNA damage repair (DDR) pathways include direct repair, base excision repair, nucleotide excision repair, mismatch repair (MMR), homologous recombination (HR), non-homologous end joining (NHEJ), and the Fanconi anemia repair pathway, with each of these pathways directed at specific types of DNA damage [1].

**Figure 1:**
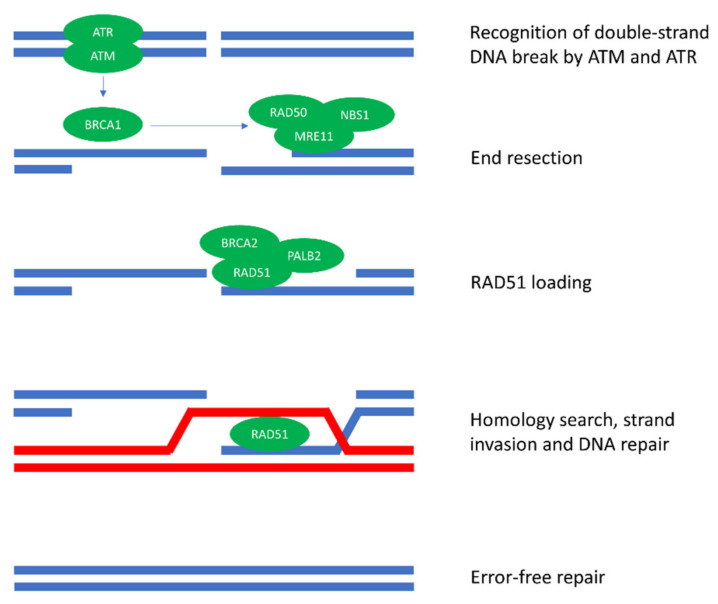
Homologous recombination repair (HR). HR commences with the recognition of double-strand DNA breaks by ATM and ATR. ATM and ATR activate BRCA1, which plays a key role in the recruitment of repair proteins needed for DNA end resection. DNA end resection generates a long 3′ single-strand DNA tail that can invade the homologous DNA strand (sister chromatid)**.** After end resection, RAD51 is loaded on the single-strand DNA tail with the help of BRCA2 and PALB2. The strand then invades the homologous DNA strand where the actual DNA repair is performed. Since a sister chromatid is used as a template for DNA repair, HR is considered a relatively error-free process. Of note, besides the proteins depicted here, many other proteins are involved in HR.

In cancer, DDR is frequently disrupted, leading to genomic instability. One of the pathways that is regularly altered in cancer is HR. HR is an important pathway for the repair of double-strand DNA breaks (DSBs) during the S and G2 phase of the cell cycle, i.e., after DNA replication has occurred. HR is considered a relatively error-free process because it uses an intact sister chromatid to guide DNA repair (Figure 1). HR deficiency (HRD) leads to enhanced reliance on alternative pathways involved in DSB repair, i.e., classical NHEJ, alternative end joining, and single-strand annealing [2,3]. These pathways repair DSBs without a homologous DNA template, resulting in characteristic genomic scars across the genome [4,5].

Two well-known genes that play an important role in HR are *BRCA1* and *BRCA2* (Figure 1). Germline pathogenic *BRCA* variants have long been recognized for their role in cancer susceptibility, increasing the risk of breast, ovarian, prostate, and pancreatic cancers [6,7]. Nevertheless, *BRCA* mutations can also arise in tumors of patients without pathogenic germline variants. Deleterious variants in *BRCA1* or *BRCA2*, either germline or somatic, are most frequently observed in ovarian cancer (13.8%), castrate-resistant prostate cancer (11.8%), and breast cancer (6.8%), but can occur in many other cancer types as well [8,9]. In addition to *BRCA1* and *BRCA2*, many other genes play a direct or indirect role in HR. Nevertheless, the exact implications of aberrations in other HR-related genes for the functionality of the HR pathway are largely unclear [10].

HRD tumors respond differently to anti-neoplastic agents as compared to non-HRD tumors. *BRCA*-mutated ovarian, breast, and prostate cancers have been described to be more sensitive to DNA-damaging chemotherapy, i.e., platinum chemotherapy [11,12,13], or poly(ADP-ribose) polymerase (PARP) inhibitors [9,14,15]. Nevertheless, patients ultimately develop resistance to these therapies. There is a need for more effective and durable treatment strategies.

In the last decade, immune checkpoint inhibitors have been registered for the treatment of several cancer types. Currently approved agents target programmed cell death protein 1 (PD-1), its ligand PD-L1, or cytotoxic T-lymphocyte-associated protein 4 (CTLA-4). While checkpoint inhibitors induce responses and improve overall survival (OS) in various types of cancers, a long-term benefit is observed in only a minority of patients. At present, it is largely unclear which patients will benefit from it. A role for DDR defects in selecting patients for immunotherapy has been suggested. MMR-deficient tumors, which are characterized by a hypermutated genome and instability of DNA repeat regions, have been shown to be responsive to checkpoint inhibitors, independent of tumor type [16]. Other DDR defects, particularly those leading to HRD, may also render tumors more susceptible for checkpoint inhibitors.

The first part of this review describes how HRD might mechanistically affect antitumor immunity. In the second part, the current evidence for an association between HRD and tumor-infiltrating immune cells is summarized and an overview is given of available clinical data on the efficacy of checkpoint inhibitors in HRD tumors. We focus on tumors with BRCA inactivation, as the functional implications of other HR-related genes remain uncertain and studies considering genome-wide HRD signatures are scarce. Finally, we describe the evidence for synergism between checkpoint inhibitors and PARP inhibitors in BRCA-inactivated tumors.

## 2. How HRD Influences Antitumor Immunity

### 2.1. Tumor Mutational Burden and Neoantigen Load

**Figure 2:**
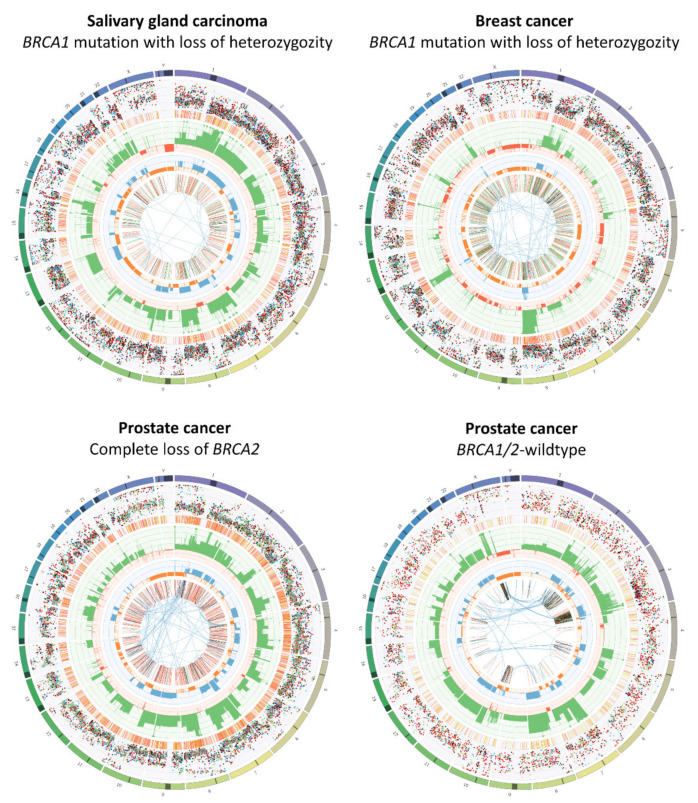
The genomic landscape of *BRCA*-mutated and *BRCA*-wildtype tumors. The depicted circos plots were generated using whole genome sequencing data of CPCT-02 study participants treated in the Radboudumc. Results of the CPCT-02 have previously been published elsewhere [30]. The outer first circle shows the chromosomes. The darker areas represent large gaps in the human reference genome, i.e., regions of centromeres. The second circle shows all somatic single nucleotide variants (SNVs) across the genome. Tumor purity-adjusted allele frequencies are scaled from 0% to 100%. SNVs are colored according to the type of base change in concordance with coloring used in previous literature [31]. Base substitutions that frequently occur in HRD are displayed in blue (C > A) and black (C > G). The third circle depicts short insertions (yellow) and deletions (red, indels). The fourth circle shows all copy number changes. Losses and gains are indicated in red and green, respectively. The scale ranges from 0 (complete loss) to 6 (high-level gains). Absolute copy numbers above 6 are indicated by a green dot on the diagram. The fifth circle represents the observed minor allele copy numbers. The scale ranges from 0 to 3, with losses (<1) shown in orange and gains (>1) shown in blue. The innermost circle displays the structural variants within or between the chromosomes. Translocations are indicated in blue, deletions are indicated in red, insertions are shown in yellow, tandem duplications are indicated in green, and inversions are shown in black. The figure shows that *BRCA*-mutated tumors generally have higher numbers of SNVs, small indels, deletions, and tandem duplications (the latter is only more frequent in *BRCA1*-mutated tumors).

To avoid autoimmunity, the immune system discriminates self-antigens from non-self-antigens. Due to mutations in protein-encoding genes, tumors may express aberrant antigens, known as neoantigens. These neoantigens may be recognized by the immune system as non-self, thereby generating an adaptive immune response, resulting in the selective elimination of cancer cells. HRD tumors exhibit a unique mutational signature, characterized by base-substitution signature 3 (enriched in C > G substitutions) and 8 (enriched in C > A substitutions) as well as an elevated number of small deletions (indels) with flanking microhomology (Figure 2) [5,17]. Although tumor mutational burden (TMB) in HRD tumors is generally not as high as in MMR-deficient tumors, HRD tumors have consistently been described to have a higher TMB as compared to HR-proficient tumors [18,19,20,21,22,23,24,25]. For instance, among two cohorts of breast cancer patients, the TMB was 2.0 to 2.6 times higher in patients with a *BRCA1* or *BRCA2* mutation as compared to those without a *BRCA* mutation [22]. Across several types of cancers, high TMB has been associated with improved outcomes of checkpoint inhibitor therapy [26,27,28,29]. A recent analysis among 1662 patients with various cancer types showed that high TMB, defined as the highest 20% of each tumor type, was associated with improved OS (hazard ratio = 0.61, *p* = 1.3 × 10^–7^) [29].

Although a higher TMB increases the likelihood of the formation of neoantigens that are able to induce an immune response, not all non-synonymous mutations give rise to immunogenic neoantigens. Neoantigens are presented on the surface of cancer cells by major histocompatibility complex (MHC) molecules. The immunogenicity of neoantigens depends on its binding affinity to the patients’ MHC molecule. Several tools have been developed to predict neoantigen load, by inferring the MHC-peptide binding affinity from sequencing data. Like TMB, a high neoantigen load has been associated with checkpoint inhibitor efficacy [27,32,33]. The neoantigen load has been described to be 2-fold to 3-fold higher in *BRCA*-mutated tumors as compared to *BRCA* wild-type tumors [22].

### 2.2. Copy Number Variations

**Figure 3:**
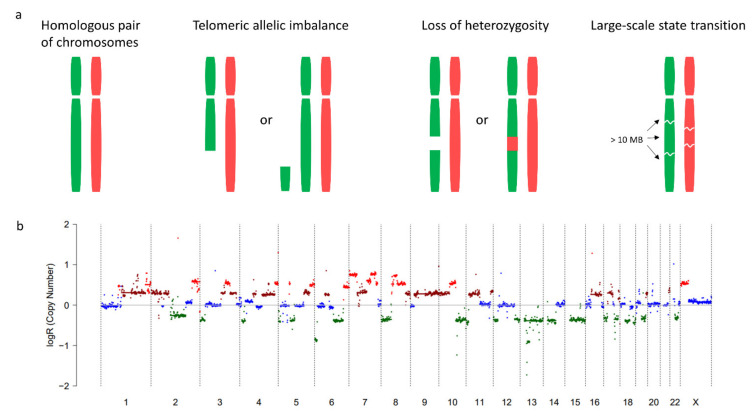
Telomeric allelic imbalance (TAI), loss of heterozygosity (LOH), and large-scale state transitions (LSTs). (**a**) Genomic scars characteristic for homologous recombination repair deficiency (HRD) include TAI, LOH, and LSTs. Allelic imbalance is the imbalance in paternal and maternal alleles with or without changes in the overall copy number of that region. Characteristic for HRD is AI at the telomeric end of a chromosome (TAI). LOH refers to the situation where one of the two alleles that was originally present in the cell is lost. LSTs are defined as chromosomal breaks between adjacent regions of at least 10 mb. (**b**) CNV profile of an HRD tumor. The plot was generated using whole genome sequencing data of a CPCT-02 study participant treated in the Radboudumc [30]. Dots represent regions of 10 mb. As LSTs lead to copy number changes, dots with a log ratio ≠ 0 indicate LSTs.

The genomic instability of HRD tumors not only leads to a higher TMB, but also to large structural changes that result in a gain or loss of part of a chromosome. Research in breast and ovarian cancer identified three genomic signatures characteristic for HRD, which may result in copy number variations (CNVs). These include telomeric allelic imbalance (TAI) [34], loss of heterozygosity (LOH) [35], and large-scale state transitions (LST) [36] (Figure 3). Furthermore, the presence of ~10 kb duplications is specific for *BRCA1*-mutated tumors but not for other HRD tumors (Figure 2) [5,37].

While little is known about the link between TAI, LOH, and LSTs and antitumor immunity, a relation has been suggested between immunity and the fraction of the genome altered by CNVs (CNV fraction). A large-scale analysis, including 9125 samples of 33 cancer types, demonstrated that the total number of TAI, LOH, and LSTs positively correlates with the CNV fraction, indicating that HRD tumors generally have a higher CNV fraction [10]. A pan-cancer analysis of The Cancer Genome Atlas (TCGA) data showed that the CNV fraction negatively correlates with cytotoxic immune signatures, i.e., genes specific for cytotoxic CD8^+^ T cells and natural killer cells [38]. The relation between the CNV fraction and the clinical outcome following treatment with anti-CTLA-4 was assessed in two independent cohorts of melanoma patients (*n* = 110 and *n* = 64). In both cohorts, a high CNV fraction was predictive of poor survival following treatment with anti-CTLA-4 (hazard ratio = 2.2, *p* = 0.0004 and hazard ratio = 2.3, *p* = 0.03, resp.) [38]. Another study that assessed the relationship between the CNV fraction and response to anti-PD-(L)1 in 248 non-small cell lung cancer (NSCLC) patients showed an inverse relation between the CNV fraction and response to checkpoint inhibitors (*p* = 0.02) [39].

There is increasing evidence that CNVs play a critical role in tumorigenesis [38]. Nevertheless, it is largely unclear why a high CNV fraction is associated with low cytotoxic immune signatures and a poor response to checkpoint inhibitors. It has been suggested that CNVs induce proteotoxic stress and, thereby, impair the signals needed to attract cytotoxic immune cells [38]. An alternative hypothesis is that patients with a high CNV fraction more frequently harbor loss of tumor suppressor genes or amplification of driver genes that have been implicated in antitumor immunity, such as PTEN loss [40] or MYC amplification [41]. In addition, loss of HLA loci, which encode MHC (or HLA) molecules, has been suggested to provide an advantage to cancers and allow for the outgrowth of subclones with an increased neoantigen load [42].

### 2.3. STING Pathway

Apart from the distinct genomic aberrations found in HRD tumors, the accumulation of DNA damage in these tumors may also affect their immunogenicity. Defects in the HR pathway have been associated with activation of the stimulator of interferon genes (STING) pathway in dendritic cells [43] and tumor cells [44]. In this pathway, cytosolic DNA is sensed by cyclic GMP-AMP synthase (cGAS), leading to activation of STING and enhanced transcription of type I interferon (IFN) genes [43]. Type I IFNs have immunostimulatory functions and play a role in promoting cross-presentation of antigens by dendritic cells, thereby, enhancing antigen-specific T cell responses [45]. Preclinical research has shown that activation of the STING pathway by STING agonists induces immune-mediated tumor regression [46,47].

There is accumulating evidence that cytosolic DNA is increased in DDR-deficient cells and that this leads to altered STING pathway activity. Research in mice deficient for *ATM* and patients with congenital *ATM* deficiency demonstrated that loss of *ATM*, which is a DNA damage sensor, is associated with enhanced type I IFN production, which results from the accumulation of cytosolic DNA and activation of the STING pathway [48]. In *BRCA1*-mutated breast cancer cells, increased cytosolic DNA levels and enhanced STING pathway activation have also been observed [49]. Additionally, in HRD breast cancer cell lines and in vivo models, treatment with PARP inhibitors, which increases DSB formation, enhanced STING pathway activation and resulted in the recruitment of immune cells [44].

In summary, the slightly increased TMB and the STING-mediated upregulation of type I IFN genes observed in HRD tumors suggest that these tumors might be more immunogenic. The higher number of CNVs, on the other hand, might suppress antitumor immunity. This raises the question which of the mechanisms predominates in driving the immunogenicity of HRD tumors.

## 3. The Tumor Immune Microenvironment in BRCA-Inactivated Tumors

A comparison of the immune infiltrate between HRD and HR-proficient tumors could provide important insights into the immunogenic consequences of HRD. While a uniform definition of immunogenicity is lacking, a high number of tumor-infiltrating lymphocytes (TILs), especially of CD8^+^ T cells, is commonly considered indicative of immunogenicity [50]. A more detailed description of the different immune cell subsets and checkpoint molecules discussed in this paragraph is given in the Box 1. In this section, we focus on differences in the immune infiltrate between *BRCA*-inactivated and *BRCA* wild-type tumors.

### 3.1. Breast Cancer

Several studies in breast cancer suggest an association between *BRCA* mutation status and increased immune cell infiltration, especially for the *BRCA1*-mutated tumors. Nolan and colleagues evaluated the presence of TILs in triple negative breast cancer (TNBC) patients with (*n* = 29) and without (*n* = 64) pathogenic germline *BRCA1* variants. Higher numbers of TILs were observed in *BRCA1*-mutated tumors as compared to *BRCA1* wild-type tumors. The immune infiltrate in *BRCA1*-mutated tumors consisted of cytotoxic (CD8^+^) and helper (CD4^+^) T cells, with a low frequency of regulatory T cells (Tregs) [21]. In accordance with this, a large-scale analysis in 1269 breast cancer patients revealed that low protein expression of BRCA1 was associated with high numbers of CD8^+^ TILs as compared to patients with normal BRCA1 expression [51]. While the previously mentioned studies focused on *BRCA1*-mutated tumors, others also took *BRCA2* mutation status into account. Kraya and colleagues found that cytolytic activity, defined as the mean expression of *PRF1* and *GZMA,* was higher in patients with a *BRCA*-mutated tumor (48 *BRCA1*-mutated, 41 *BRCA2*-mutated) compared to patients with an HR-proficient tumor (*n* = 652), with no difference between *BRCA1*-mutated tumors and *BRCA2*-mutated tumors [23]. Wen and colleagues, on the other hand, showed that only pathogenic *BRCA1* but not *BRCA2* variants were associated with a higher number of activated CD4^+^ and CD8^+^ T cells using transcriptome data of the Wellcome Sanger Institute and TCGA (*n* = 1418, 78 *BRCA1*-mutated and 53 *BRCA2*-mutated) [22].

While several studies suggest that *BRCA1*-mutated breast cancers have increased immune cell infiltration, there are also numerous studies that did not find any association between *BRCA* mutation status and immune cell infiltration [11,19,52,53]. Further complicating the interpretation of the results, a recent study indicates that *BRCA1*-mutated tumors (*n* = 17) have a more immunosuppressed tumor microenvironment as compared to *BRCA1* wild-type tumors, as evidenced by higher expression of immunoregulatory and suppressive genes [54]. Interestingly, this was not the case for *BRCA2*-mutated tumors (*n* = 18). The authors observed lower numbers of SNVs and indels and higher CNV fractions in *BRCA1*-mutated tumors as compared to *BRCA2*-mutated tumors and suggest that these genomic differences may contribute to the observed differences in immunogenicity.

### 3.2. Ovarian Cancer

Most studies in ovarian cancer have reported increased TILs and immune checkpoint expression in *BRCA*-mutated tumors. In a cohort of 53 patients with serous ovarian cancer (29 *BRCA1*-mutated, 8 *BRCA2*-mutated, 16 HR-proficient), *BRCA*-mutated tumors exhibited increased CD3^+^ and CD8^+^ T cells as compared to HR-proficient tumors. PD-1 and PD-L1 expression on tumor-infiltrating immune cells was also higher in *BRCA*-mutated tumors, but no significant difference was observed in PD-L1 expression on tumor cells or the number of B cells [55]. In line with these findings, a study in 40 patients, including 18 patients with a *BRCA1*-mutated tumor (*n* = 9) or a tumor with epigenetic loss of *BRCA1* (*n* = 9), demonstrated that intraepithelial CD8^+^ TILs were more frequently observed in tumors with *BRCA1* abnormalities (94.4% vs. 57.9%) [56]. Additionally, a study among 158 ovarian cancer patients (37 *BRCA*-mutated, 121 *BRCA*-wildtype) showed that *BRCA*-mutated tumors had significantly higher levels of *PD-1* and *PD-L1* mRNA as compared to *BRCA* wild-type tumors [57]. Finally, in a study among 103 patients with serous ovarian cancer (21 *BRCA1*-mutated, 10 *BRCA2*-mutated, 21 *BRCA1* methylation, and 51 no *BRCA* loss), *BRCA*-mutated tumors tended to be more frequently infiltrated by CD8^+^ T cells (92.9%) as compared to tumors with *BRCA1* methylation (76.2%) or no *BRCA* loss (73.9%, *p* = 0.057) [58]. In contrast to the breast cancer studies that are described above, the ovarian cancer studies analyzed *BRCA1*-mutated and *BRCA2*-mutated tumors as a single group, making it impossible to evaluate the contribution of the individual genes.

Like in breast cancer, there are also a few studies in ovarian cancer that do not support an association between *BRCA*-inactivation and immune cell infiltration. In an immunohistochemistry study including 48 patients with serous ovarian cancer and known germline *BRCA* mutation status (4 *BRCA1*-mutated, 8 *BRCA2*-mutated, 36 *BRCA*-wildtype), no association was found between germline *BRCA* mutation status and the infiltration of CD8^+^ T cells or Tregs or checkpoint expression (PD-L1 or LAG-3) [59]. Furthermore, analyses of transcriptome data of the TCGA yielded conflicting results [19,60].

### 3.3. Prostate Cancer

In an attempt to better understand the impact of *BRCA2* mutations on the immune phenotype of prostate cancer, Jenzer and colleagues performed immunohistochemistry and T-cell receptor (TCR) sequencing in nine *BRCA2*-mutated and nine *BRCA* wild-type, hormone-sensitive prostate cancers. No difference was observed in the number of T cell clones or TCR clonality. In *BRCA2*-mutated tumors, however, the ratio between intratumoral and stromal CD4^+^ T cells, CD8^+^ T cells and Tregs was higher as compared to *BRCA2* wild-type tumors. Although the location of the T cells does not inform us about the antitumor activity of these cells, the closer proximity to the tumor cells does suggest a more active immune response [61].

Box 1 **Immune Cell Subsets and Immune Checkpoints.**

- T cells are key players in antitumor immunity. They are able to selectively target cancer cells following recognition of non-self-antigens. T cells, characterized by the expression of CD3, can be subdivided into cytolytic T cells (CD8^+^), helper T cells (CD4^+^), and regulatory T cells (CD4^+^FoxP3^+^). While cytolytic T cells and helper T cells play an important role in tumor immunosurveillance, regulatory T cells suppress antitumor immunity. Studies in various cancer types indicate that high intratumoral CD8^+^ T cell density is associated with favorable outcomes to checkpoint inhibitor therapy [62,63]. Nevertheless, the sole presence of CD8^+^ T cells does not necessarily indicate an active immune response. Immune activity can be inhibited by a lack of antigen presentation or by the presence of immune suppressive cells, cytokines, or inhibitory checkpoint molecules.
- B cells play a major role in antibody-mediated immunity. Although their role in antitumor immunity is not completely understood, recent data suggest that B cells play a role in antitumor immunity and promote checkpoint inhibitor efficacy [64].
- Natural killer cells are innate immune cells with a cytolytic function.
- Checkpoint molecules play an important role in regulating immune responses. PD-L1, PD-1, and LAG-3 are all inhibitory checkpoint molecules. Activation of these checkpoints suppresses immune cell activation. In some cancer types, PD-L1 expression is associated with a response to PD-(L)1 inhibitors [65]. In NSCLC and urothelial cancer, PD-L1 expression is used for treatment stratification.

### 3.4. Summary

Although several studies in various cancer types indicate that *BRCA*-inactivated tumors have more dense immune infiltrates, current data is inconclusive. There are several possible explanations for these heterogeneous results. First, study results might have been biased due to the presence of sporadic cancers in the *BRCA*-mutated group. In most studies the *BRCA*-mutated group was not limited to patients with biallelic *BRCA* inactivation. Across cancer types, only 89% of patients with a germline *BRCA1* variant and 79% of patients with a *BRCA2* variant have a tumor with complete loss of the wild-type allele [30]. Besides the presence of sporadic cancers in the *BRCA*-mutated group, there might also have been HRD tumors in the *BRCA*-wildtype group as HRD can also arise from mutations in other HR genes or promoter hypermethylation [5,17,66]. Recently, genome-wide, mutational scar-based scores have been developed for the assessment of HRD, such as HRDetect [17] and CHORD [5]. Up to 45% of cancer patients with an HRD tumor according to CHORD do not have an event in *BRCA1* or *BRCA2* [5]. Unfortunately, currently available data on the association of HRD and immune cell infiltration has focused on the *BRCA* mutation status and did not take genome-wide HRD signatures into account. Finally, an explanation for the inconsistent results might be the heterogeneity of HRD tumors. The immunogenicity of the HRD tumor might differ depending on the degree of genomic instability and the genomic regions where alterations have occurred. It is conceivable, for example, that amplification or loss of driver genes involved in immune suppression might hamper antitumor immunity despite higher TMB and STING pathway activation. While evidence on this subject is currently limited, it is plausible that only those tumors with a high number of SNVs and indels and a low CNV fraction are more immunogenic. In support of this, a study in breast cancer patients described a negative association between T cell infiltration and the degree of LOH, TAI, and LSTs within the *BRCA*-mutated subgroup [23].

## 4. Checkpoint Inhibitor Therapy in BRCA-Inactivated Tumors

### 4.1. Tumor Types with Low Sensitivity to Checkpoint Inhibitor Monotherapy

Although checkpoint inhibitors have greatly improved clinical outcomes in some cancers, checkpoint inhibitors have had limited success in many other tumor types so far. These tumor types include breast cancer, ovarian cancer, and prostate cancer, i.e., tumor types where HRD occurs rather frequently. Although checkpoint inhibitors are not beneficial for the entire group of breast cancer, ovarian cancer, or prostate cancer patients, selected subgroups may benefit. HRD has been suggested to function as a biomarker to select patients for checkpoint inhibitor therapy.

#### 4.1.1. Breast Cancer

Most research on checkpoint inhibitors in breast cancer has focused on patients with TNBC, a subgroup that is enriched for *BRCA1* mutations [67]. Checkpoint inhibitors have shown modest activity in breast cancer, when used as a single agent [68]. Nevertheless, the combination of the PD-L1 inhibitor atezolizumab with the chemotherapeutic agent nab-paclitaxel has been shown to improve median OS as compared with nab-paclitaxel alone in PD-L1^+^ TNBC (25.0 to 15.5 months) [69]. Preclinical studies suggest that *BRCA2*-mutated but not *BRCA1*-mutated breast cancers are responsive to treatment with checkpoint inhibitor monotherapy [21,54]. However, clinical studies supporting this are lacking. Data from clinical trials suggest that *BRCA*-mutated TNBCs are not more susceptible to treatment with atezolizumab plus nab-paclitaxel [70]. Furthermore, *BRCA1*-like, genomic copy number profiles appear to be negatively associated with response to PD-1 blockade in TNBC [71] (Table 1).

#### 4.1.2. Ovarian Cancer

Clinical trials on the efficacy of single-agent checkpoint inhibitor therapy in ovarian cancer have reported response rates around 10% [72,83]. Due to these low response rates, checkpoint inhibitors have not (yet) been approved for the treatment of ovarian cancer, apart from the subset of patients with MMR-deficient tumors [16]. Data on the efficacy of checkpoint inhibitors in HRD ovarian cancer is limited. A phase Ib trial on the efficacy of PD-L1 inhibitor avelumab reported only one objective response among eight patients with a pathogenic germline *BRCA* variant (12.5%). This response rate was very similar to that observed in *BRCA*-wildtype patients (7.9%) [72]. A case series, on the other hand, described very promising responses to PD-1 inhibitor nivolumab among six patients with germline *BRCA* mutations and recurrent ovarian (*n* = 5) or fallopian tube (*n* = 1) cancer. Four out of six patients achieved an objective response, including three complete responses [73]. Although it is possible that checkpoint inhibitors are more effective in patients with HRD ovarian cancer, the low response rates to checkpoint inhibitors together with the high frequency of HRD in ovarian cancer (up to 50%) indicates that checkpoint inhibitors are not effective in all patients with HRD ovarian cancer [5].

#### 4.1.3. Prostate Cancer

Checkpoint inhibitor monotherapy has not been able to improve the clinical outcome in unselected patients with castrate-resistant prostate cancer (CRPC). Only 3–5% of CRPC patients achieve an objective response to anti-PD-1 [75]. The response rate to combination therapy with ipilimumab and nivolumab appears to be higher. Yet, still only 10–26% of patients achieve an objective response [84]. Exploratory biomarker analyses in clinical trials have suggested that HRD tumors might be more sensitive to checkpoint inhibitors. In the KEYNOTE-199, the objective response rate to pembrolizumab was 11% in patients with *BRCA1*-mutated, *BRCA2*-mutated, or *ATM*-mutated tumors and 3% in patients without mutations in HR-related genes [75]. In the CheckMate 650, 50% of patients with an HRD tumor and only 22.6% of patients with HR-proficient tumors responded to combination therapy [76]. Importantly, in the latter study, the authors used a broad definition of HRD, including not only *BRCA,* but also *ATM, CDK12*, and *FANCA* alterations. Only tumors with *BRCA2* and *FANCA* mutations responded to therapy. Although promising, one should keep in mind that the number of patients in this trial were low (Table 1).

### 4.2. Tumor Types Responsive to Checkpoint Inhibitor Monotherapy

Biomarkers that enrich for response to checkpoint inhibitors may also have great utility in tumor types where checkpoint inhibitors are already part of standard care. In these tumor types, biomarkers may guide the treatment sequence and may help decide between checkpoint inhibitors monotherapy or combinational treatment strategies. Therefore, it is important to know how HRD affects checkpoint inhibitor sensitivity in these tumors.

#### 4.2.1. Urothelial Cancer

In urothelial cancer, PD-(L)1 inhibitors are currently mostly used as second-line treatment for patients with metastatic disease who progressed on platinum-based chemotherapy [85]. Recent trials investigating the efficacy of checkpoint inhibition in the first-line setting showed no survival benefit for checkpoint inhibitors over chemotherapy in the overall population [86,87]. Nevertheless, first-line therapy with checkpoint inhibitors may be very effective in selected subgroups. A study of 60 patients with advanced urothelial cancer showed that 80% of patients who had tumors with a deleterious DDR alteration had an objective response to anti-PD-(L)1, whereas responses were seen in only 18.8% of patients without DDR alterations. Importantly, only three out of fifteen patients with deleterious DDR alterations harbored tumor mutations in *BRCA1* or *BRCA2* and no information is provided on the responses of these three patients. Other DDR mutations included *ATM*, *POLE*, *ERCC2*, *FANCA*, and *MSH6* [78].

#### 4.2.2. Other Cancer Types

The incidence of HRD in other tumor types where checkpoint inhibitors are part of standard care is very low. This includes melanoma, non-small cell lung cancer, and renal cell carcinoma [5]. There have been a few reports on the association between *BRCA* mutations and checkpoint inhibitor efficacy in these tumors. Nevertheless, none of these studies reported on the zygosity status or pathogenicity of the identified mutations, making it difficult to interpret the results. These data are summarized in Table 1.

### 4.3. Pan-Cancer Analyses

A recent, large-scale, pan-cancer analysis in 1661 patients treated with checkpoint inhibitors demonstrated a significantly longer OS in patients with tumors with a mutation in an HR-related gene [82]. Patients were treated with antibodies targeting CTLA-4 (9%), PD-(L)1 (76%), or both (16%). The authors distinguished between HR-related genes (*ARID1A, BLM, BRCA2, MRE11, NBN, RAD50, RAD51/B/D, RAD52, RAD54L, XRCC2*) and DNA checkpoints (including, among others, *BRCA1, ATM*, *CHEK1*, and *CHEK2*). Patients with tumor mutations in HR-related genes had significantly longer OS as compared to those without these mutations, independent of tumor type or TMB (41 months vs. 16 months, adjusted hazard ratio = 1.39, 95% CI 1.15–1.70, *p* < 0.001). In contrast to the HR-related genes, the DNA checkpoints were not associated with OS after adjustment for TMB and tumor type. The most frequently mutated HR-related genes were *ARID1A* (11.4%) and *BRCA2* (5.6%). Mutations in *BRCA2* as well as most other HR-related genes with an incidence of least 1% (*ARID1A*, *RAD50, RAD51B*, and *MRE11*) were individually associated with longer OS. In a cohort of patients not treated with checkpoint inhibitors, mutations in HR-related genes were associated with worse OS (HR 0.86, 95% CI 0.78–0.95, *p* = 0.003), suggesting that mutations in the designated HR-related genes have predictive value for response to checkpoint inhibitors rather than a prognostic value. Despite the retrospective character, the broad definition of HR-related genes, and the fact that the observed non-synonymous mutations were not assessed for their functional effects, this large scale analysis supports the idea that mutations in *BRCA2* and other genes with a direct or indirect role in HR render tumors more susceptible for treatment with checkpoint inhibitors.

In line with these findings, another large study of 2185 patients with various cancer types also suggests higher sensitivity of *BRCA2*-mutated tumors to checkpoint inhibitors. Included patients were treated with anti-PD-(L)1, CTLA-4, or a combination of both. In total, 67 patients harbored a pathogenic germline or somatic variant in *BRCA2* and 28 in *BRCA1*. Zygosity status was not assessed. In univariate analysis, *BRCA2* but not *BRCA1* mutations were associated with improved OS after checkpoint inhibitor therapy. The correlation between *BRCA2* mutations and OS remained significant after controlling for tumor type and TMB (HR 0.50, 95% CI 0.30 – 0.83, *p* = 0.008). It is difficult to make a direct comparison between *BRCA2*-mutated and *BRCA1*-mutated tumors as the distribution of these mutations differs across cancer types and the correlation between the *BRCA1* mutation status and OS was not controlled for tumor type. Nevertheless, the data suggest that patients with *BRCA2*-mutated tumors are more susceptible for treatment with checkpoint inhibitors.

### 4.4. Summary

There is evidence from two large pan-cancer analyses suggesting that checkpoint inhibitors are more effective in patients with *BRCA2*-mutated tumors. Data from other studies is limited by the small sample size, the lack of information on the pathogenicity of the identified mutations and zygosity status, and/or the broad definition of HR-related genes. All clinical data is summarized in Table 1. Prospective studies are needed to validate the findings of the two large pan-cancer trials and to provide more insight into HRD-associated hallmarks associated with responses to checkpoint inhibitors. As evident from the low response rates to checkpoint inhibitors in ovarian cancer, where HRD occurs in up to 50% of patients, it is clear that not all patients with HRD will respond to checkpoint inhibitors. Additional factors, such as the presence of a *BRCA1*-type or *BRCA2*-type HRD signature, the TMB, and the CNV fraction, might influence sensitivity to checkpoint inhibitors in these tumors. Phase II trials in patients with advanced solid tumors (ClinicalTrials.gov Identifier: NCT03428802) and metastatic CRPC (ClinicalTrials.gov Identifier: NCT04717154) have recently been initiated to study the efficacy of checkpoint inhibitors in HRD tumors. If (a subset) of HRD tumors prove to be more sensitive to checkpoint inhibitor therapy, this will have important implications for treating patients with HRD tumors.

## 5. Combining Checkpoint Inhibitors with DNA-Damaging Agents

Among various cancer types, *BRCA* mutations have been found to sensitize tumors to treatment with PARP inhibitors. In light of the presumed increased immunogenicity of HRD tumors, it is important to discover whether treatment strategies that combine checkpoint inhibitors with PARP inhibitors might be effective in treating these cancers. PARP inhibitors currently have a role in the treatment of ovarian cancer, pancreatic cancer, HER2-negative breast cancer, and CRPC [9].

PARPs play a critical role in the repair of single-strand DNA breaks (SSBs). PARP inhibitors use two distinct mechanisms. First, PARP inhibitors prevent the repair of SSBs, which will eventually lead to the accumulation of DSBs. In HRD tumors, this is lethal because DSBs cannot be repaired by the HR pathway. Second, PARP inhibitors trap PARP proteins at the site of SSBs, thereby preventing DNA replication [88]. PARP inhibitors have been suggested to have an immunomodulatory effect. In preclinical studies, upregulation of PD-L1 was observed during PARP inhibition [89,90]. In addition, PARP inhibitors have been shown to promote STING pathway activation [91]. Therefore, combining checkpoint inhibitors and PARP inhibitors might be a promising treatment strategy for HRD cancers.

In breast and ovarian cancer mouse models, PARP inhibitors and checkpoint inhibitors were found to have synergistic activity [89,91,92,93]. Clinical data supporting the superiority of combination therapy over PARP inhibitor monotherapy, however, is lacking. A recent phase I/II clinical trial evaluated the safety and efficacy of combination therapy with PD-L1 inhibitor durvalumab and PARP inhibitor olaparib in 34 patients with germline *BRCA*-mutated, metastatic breast cancer (16 × *BRCA1*, 18 × *BRCA2*). Patients were allowed to have received a maximum of two previous lines of chemotherapy. The safety profile of the combination appeared similar to durvalumab or olaparib monotherapy. Objective responses were observed in 63% of patients. The median duration of the response was 9.2 months (95% CI 5.5–13.1), the median progression-free survival (PFS) was 8.2 months (95% CI 4.6–11.8), and the median OS was 21.5 months (95% CI 16.2–25.7) [94]. These results are comparable with the efficacy of olaparib monotherapy in germline *BRCA*-mutated breast cancer, as reported in the OlympiAD trial [15].

The efficacy of the PD-1 inhibitor pembrolizumab and the PARP inhibitor niraparib in recurrent ovarian cancer was evaluated in 62 patients. In this phase I/II trial, 11 patients (18%) harbored a mutation in *BRCA1* (*n* = 9) or *BRCA2* genes (*n* = 2). The combination induced an objective response in 18% of patients and disease control in 65%. Median PFS was 3.4 months (95% CI, 2.1–5.1 months), median duration of the response was not reached (ranging from 4.2 to ≥14.5 months), and OS data was not yet mature. Exploratory analyses indicated that the combination of niraparib and pembrolizumab resulted in anti-tumor activity regardless of the *BRCA* mutation status or HRD status [95].

In conclusion, although preclinical data suggest that PARP inhibitors and checkpoint inhibitors might work synergistically, clinical data confirming this hypothesis is lacking. Fortunately, several phase I-III clinical trials investigating the combination of PARP inhibitors and checkpoint inhibitors in HRD tumors are underway (ClinicalTrials.gov Identifiers: NCT02571725, NCT03101280, NCT02849496, NCT03330405, NCT04508803, NCT04493060, NCT03824704, NCT02953457, NCT03834519).

## 6. Conclusions

In summary, the modest increase in TMB and the STING-mediated upregulation of type I IFN genes observed in HRD tumors imply that HRD tumors are more immunogenic. Yet, data regarding the association between *BRCA* mutations and immune cell infiltration is inconsistent. Two large pan-cancer trials suggest that checkpoint inhibitors might be more effective in *BRCA2*-mutated tumors, but this requires further validation. Factors accounting for the differing study results may include differences in study populations, infrequent assessment of the zygosity status, HRD among BRCA-wildtype patients, and heterogeneity within the HRD group inherent to the genomic instability of these tumors. Prospective studies are needed to test whether HRD or HRD-associated hallmarks can function as biomarkers to select patients for treatment with checkpoint inhibitors, either as a single-modality treatment or in combination with PARP inhibitors.

## Figures and Tables

**Table 1:** The efficacy of checkpoint inhibitors in HRD tumors.

Reference	Tumor Type	*N*		Genes	Treatment	Results ^1^
		Total	Mut			
[70]	TNBC	612	89	Pathogenic germline or somatic *BRCA1/2* variants, zygosity status not assessed	Atezolizumab + nab-paclitaxel	PFS: hazard ratio 1.07, 95% CI 0.77–1.49 OS: hazard ratio 1.07, 95% CI 0.71–1.62
[71]	TNBC	49	25	*BRCA1*-like genomic copy number profiles	Nivolumab with or without induction chemotherapy or irradiation	Lower ORR in BRCA1-like patients (*p* < 0.05)
[72]	Ovarian cancer	46	8	Pathogenic germline *BRCA1/2* variants, zygosity status not assessed	Avelumab	ORR: 12.5% (1/8) in BRCA-mut vs 7.9% (3/38) in BRCA-WT
[73]	Ovarian or fallopian tubal cancer	6	6	Pathogenic germline *BRCA1/2* variants, zygosity status not assessed	Nivolumab	ORR: 76% (3/6 CR, 1/6 PR, 2/6 PD)
[74]	Ovarian or uterine cancer	25	2	Pathogenic germline *BRCA1* variants	Atezolizumab	ORR: Both had PD
[75]	CRPC	153	19	Pathogenic homozygous *BRCA1/2* or *ATM* aberrations	Pembrolizumab	ORR: 11% (2/19) in patients with BRCA1/2 or ATM aberrations and 3% (4/124) in patients without HR aberrations
[76]	CRPC	28	5	Pathogenic homozygous aberrations in *BRCA2, ATM, CDK12*, or *FANCA*	Ipilimumab + nivolumab	ORR: 50% (3/6) in DDR-mut vs 22.6% (7/31) in DDR-WT. Of note, responding patients in the DDR group had mutations in BRCA2 or FANCA
[77]	CRPC with AR-V7 expression	15	6	Pathogenic mutations in *BRCA2* (3), *ATM* (2), *ERCC4* (1)^2^, LOH in two *BRCA2*-mut patients	Ipilimumab + nivolumab	ORR: 40% (2/5) in DDR-mut vs 0% (0/3) in DDR-WT (*p* = 0.46) PSA response: 33% (2/6) vs 0% (0/9) (*p* = 014) PFS: hazard ratio = 0.31, 95% CI 0.10–0.92, *p* = 0.01 OS: hazard ratio = 0.41, 95% CI 0.14–1.21, *p* = 0.1
[78]	Urothelial cancer	60	15	Pathogenic alterations in *BRCA1/2* (3) and other DDR genes (12; *ATM*, *POLE*, *ERCC2*, CHEK2, *FANCA*, and MSH2, *MSH6).* Zygosity status n/a	Anti-PD-(L)1	ORR: 80% (12/15) and 19% (6/32) in patients with deleterious DDR alterations and no DDR alterations, resp. PFS: Median PFS NR^3^ and 2.9 months, resp
[79]	NSCLC	44	9	*BRCA1/2* mutations. Zygosity status and pathogenicity n/a	Anti-PD-(L)1	10% and 29% of patients with and without durable benefit resp, harbored a mutation in BRCA1/2
[80]	Renal cell carcinoma	34	12	*BRCA1/2* mutations. Zygosity status and pathogenicity n/a	Anti-PD-1 alone (32) or combined with anti-CTLA-4 (2)	38% (6/16) of patients with disease control vs. 33% (6/18) of patients with PD had a mutation in an BRCA1/2
[81]	Metastatic melanoma	38	7	*BRCA2* mutations. Zygosity status and pathogenicity n/a	Anti-PD-1	BRCA2 mutation in 28% (6/21) of responders vs. 6% (1/17) of non-responders
[82]	Various solid tumors	1661	335	*ARID1 A, BLM, BRCA2, MRE11, NBN, RAD50, RAD51/B/D, RAD52, RAD54 L, XRCC2* Zygosity status and pathogenicity n/a	Anti-CTLA-4 (9%), anti-PD-(L)1 (76%), or both (16%)	OS: Median OS 41 months in HR-mut vs 16 months in HR-WT Adj hazard ratio^4^ = 1.39, 95% CI 1.15–1.70, *p* = 0.022
[54]	Various tumors	2185	95	Pathogenic somatic or germline *BRCA1* (28) or *BRCA2* (67) mutations. Zygosity statis n/a	Anti-PD-(L)1, CTLA-4 or a combination	OS BRCA1: Hazard ratio 0.76, 95% CI 0.48–1.54, *p* = 0.45 OS BRCA2: Hazard ratio 0.48, 95% CI 0.29–0.80 Adj hazard ratio^5^ = 0.50, 95% CI 0.30–0.83, *p* = 0.008

^1^ A hazard ratio <1 indicates better outcomes in patients with HRD tumors. ^2^ ERCC4 is involved in nucleotide excision repair. ^3^ Median follow up was 19.6 months. ^4^ Adjusted for high TMB, type of ICI administered, and tumor type. ^5^ Adjusted for TMB and cancer type. Abbreviations: adj = adjusted; AR-V7 = androgen receptor variant 7; 95% CI = 95% confidence interval; CRPC = castrate-resistant prostate cancer; DDR = DNA damage repair; HR = homologous recombination; LOH = loss of heterozygosity, mut = mutated; NSCLC = non-small cell lung cancer; ORR = objective response rate; OS = overall survival; PFS = progression-free survival; PD-1 = programmed cell death protein-1; PD-L1 = programmed cell death ligand 1; TNBC = triple negative breast cancer; WT = wild type.
